# Pediatric Nephrology

**DOI:** 10.1155/2012/416749

**Published:** 2012-03-22

**Authors:** Michel Fischbach, Patrick Niaudet, Franz Schaefer, Lesly Rees

**Affiliations:** ^1^Nephrology Dialysis Transplantation Children's Unit, University Hospital Hautepierre, Avenue Molière, 67098 Strasbourg, France; ^2^Service de Néphrologie Pédiatrique, Hôpital Necker-Enfants Malades, 149 Rue de Sèvres, 75015 Paris, France; ^3^Division of Pediatric Nephrology, Center for Pediatric and Adolescent Medicine, INF 430, 69120 Heidelberg, Germany; ^4^Renal Unit, Great Ormond Street Hospital for Children NHS Trust and Institute of Child Health London, London WC1N 3JH, UK

Pediatric nephrology covers a large field of medical developments, including genetics, care strategies, and renal preservation. Most of the pediatric kidney diseases are congenital, some of them familial with a precise heredity. Growing, the children will be adult patients emphasizing the importance of better knowledge by the nephrologist of this kidney disease, to optimize transition from pediatric to adult care. Therefore, this special issue is of major interest for “all.” It was only possible to do this “issue” with the partnership of many “Friends.”

**Several Topics Are “Invited Reviews Resulting from Recognized Expert Teams**

The “*Hemolytic uremic syndrome: new developments in pathogenesis and treatment*” by P. Niaudet and O. Boyer can be divided into two forms, typical and atypical HUS. Postdiarrhea HUS, that is, typical D+ (Shiga-toxin enterohemorrhagic Escherichia coli (EHEC) or Shigella), generally of good prognosis is the most common form of HUS in children accounting for 90 percent of all cases. Atypical HUS (aHUS) is a heterogeneous disorder which is responsible for only 10 percent of cases in children. Thrombotic microangiopathy may affect other organ systems, including the central nervous system, with poorer prognosis. In the past 15 years, mutations in complement regulators of the alternative pathway have been identified in almost 60% of cases, leading to excessive complement activation. Eculizumab, a monoclonal antibody binding to C5, has been used in patients with aHUS on native kidney or recurrence of aHUS after transplantation with very encouraging results.

The “*The history of cystinosis: lessons for clinical management*” by P. Goodyer is marked by a few sudden leaps forward in our understanding rather than by a sustained research effort fuelled by the larger research community. Major conceptual breakthroughs include (a) its discovery in 1903, (b) recognition of the renal Fanconi syndrome, (c) realization that tissue accumulation of cystine reflects a defective channel in the lysosomal membrane, (d) translation of this discovery to trials of cysteamine, (e) discovery of the *CTNS* gene, and (f) report of successful stem cell therapy in the cystinotic mouse.

The “*Primary hyperoxalurias*” as marked by P. Cochat et al. are inborn errors in the metabolism of glyoxylate and oxalate. Early initiation of conservative treatment, including high fluid intake, inhibitors of calcium oxalate crystallization, and pyridoxine in responsive cases, can help to maintain renal function in compliant subjects. In end-stage renal disease patients, the best outcomes have been achieved with combined liver-kidney transplantation which corrects the enzyme defect.

In the paper by J. A. Sayer et al., the “nephronophthisis” is recognized as a ciliopathy. NPHP is a recessive monogenic disorder [19], meaning that two mutations (homozygous or compound heterozygous) in a single gene are sufficient to cause disease. Thirteen genes have been identified in affected families with NPHP, and these genes currently allow 30% of cases with NPHP to be “solved” in terms of a molecular diagnosis. NPH is a leading genetic cause of established renal failure (ERF) in children and young adults.

The “*Renal mitochondrial cytopathies*” are a group of rare diseases that are characterized by frequent multisystemic involvement and extreme variability of phenotype, including renal involvement. Most frequently patients present a tubular defect that is consistent with complete De Toni-Debré-Fanconi syndrome in most severe forms. More rarely, patients present with chronic tubulointerstitial nephritis, cystic renal diseases, or primary glomerular involvement.

The *“Molecular and genetic basis of inherited nephrotic syndrome” *describes the novel concept of the glomerular filtration barrier, highly dynamic slit diaphragm proteins. The *“Nephrotic syndrome in children: from bench to treatment”* allows a review of the clinical management of NS in children. Most of the cases are steroid sensitive, but fifty percents of the latter recur frequently and necessitate a prevention of relapses by nonsteroid drugs. But, their long-term prognosis is overall good. On the contrary, steroid-resistant nephrotic syndrome (SRINS) leads often to end-stage renal failure.

The “*Recurrent focal segmental glomerulosclerosis: a discrete clinical entity*” describes that some FSGSs are caused by mutations podocyte slit diaphragm genes, that is, hereditary FSGS. Thereby, it is increasingly clear that the steroid-resistant form of FSGS that recurs in the renal allografts (R-FSGSs) constitutes a distinct clinical entity.

The “*Primary molecular disorders and secondary biological adaptations in Bartter syndrome*” describes the Bartter syndrome as a hereditary disorder that has been characterized by the association of hypokalemia, alkalosis, and the hypertrophy of the juxtaglomerular complex with secondary hyperaldosteronism and normal blood pressure. The genetic causes of Bartter syndrome primarily affect molecular structures directly involved in the sodium reabsorption at the level of the Henle loop.

The “*Acute renal replacement therapy in pediatrics*” gives out the current knowledge on the dialysis modalities: when to apply and, how to apply.

Chronic dialysis remains a life event for most of the children on end-stage renal disease despite the growing place of preemptive kidney transplantation. “*Peritoneal dialysis tailored to pediatric needs” and “Optimal hemodialysis prescription: do children need more than a urea dialysis dose?*” offer a review on the necessarily optimized dialysis strategies for children based on collaborative clinical experiences. The concept of the “adapted automatic peritoneal dialysis” is described, as well as the place for a “convective volume” to add to the limited urea dialysis dose. “Daily” hemodiafiltration appears as an optimal package to promote statural catch up growth.

**Several Topics Reflect Original Experiences**

This special issue covers several original experiences: “*Once-daily tacrolimus extended-release formulation: 1 year after conversion in stable pediatric kidney transplant recipients,*” “*The hyponatremic hypertensive syndrome in a preterm infant: a case of severe hyponatremia with neurological sequels,*” “*Depressive symptomatology in children and adolescents with chronic renal insufficiency undergoing chronic dialysis,*” “*(Pro)renin receptor in kidney development and disease,”* and *“Urinary angiotensinogen as a biomarker of nephropathy in childhood*.”

Together, the lecturers should improve their “pediatric nephrology” knowledge, from bench to bedside.

*Michel Fischbach* *Patrick Niaudet* *Franz Schaefer* *Lesly Rees*

